# Synergism in aluminum and mercury neurotoxicity

**DOI:** 10.15761/IFNM.1000214

**Published:** 2018-04-13

**Authors:** Peter N Alexandrov, Aileen I Pogue, Walter J Lukiw

**Affiliations:** 1 Russian Academy of Medical Sciences, Moscow 113152, Russia; 2 Alchem Biotek Research, Toronto ON M5S 1A8, Canada; 3 LSU Neuroscience Center, Louisiana State University Health Sciences Center, 2020 Gravier Street, Suite 904, New Orleans LA 70112, USA; 4 Department of Neurology, Louisiana State University Health Sciences Center, 2020 Gravier Street, Suite 904, New Orleans LA 70112, USA; 5 Department of Ophthalmology, Louisiana State University Health Sciences Center, 2020 Gravier Street, Suite 904, New Orleans LA 70112, USA

## Abstract

Aluminum and mercury are common neurotoxic contaminants in our environment – from the air we breathe to the water that we drink to the foods that we eat. It is remarkable that to date neither of these two well-established environmental neurotoxins (i.e. those having a general toxicity towards brain cells) and genotoxins (those agents which exhibit directed toxicity toward the genetic apparatus) have been critically studied, nor have their neurotoxicities been evaluated in human neurobiology or in cells of the human central nervous system (CNS). In this paper we report the effects of added aluminum [sulfate; Al₂(SO₄)₃] and/or mercury [sulfate; HgSO_4_] to human neuronal-glial (HNG) cells in primary co-culture using the evolution of the pro-inflammatory transcription factor NF-kB (p50/p65) complex as a critical indicator for the onset of inflammatory neurodegeneration and pathogenic inflammatory signaling. As indexed by significant induction of the NF-kB (p50/p65) complex the results indicate: (i) a notable increase in pro-inflammatory signaling imparted by each of these two environmental neurotoxins toward HNG cells in the ambient 20-200 nM range; and (ii) a significant synergism in the neurotoxicity when aluminum (sulfate) and mercury (sulfate) were added together. This is the first report on the neurotoxic effects of aluminum sulfate and/or mercury sulfate on the initiation of inflammatory signaling in human brain cells in primary culture. The effects aluminum+mercury together on other neurologically important signaling molecules or the effects of other combinations of common environmental metallic neurotoxins to human neurobiology currently remain not well understood but certainly warrant additional investigation and further study in laboratory animals, in human primary tissue cultures of CNS cells, and in other neurobiologically realistic experimental test systems.

## Introduction

Inflammation and alterations in the innate-immune response are the precursor to many human neurological diseases. A central mediator of this immune and inflammatory response is the pro-inflammatory transcription factor NF-kB (p50/p65) complex involved in the transcriptional regulation of hundreds of pro-inflammatory genes encoding cytokines, chemokines, immuno-receptors, cell adhesion molecules and pro-inflammatory microRNAs [1–5]. While the significant up-regulation of NF-kB (p50/p65) complex is not immediately lethal to the cell, its appearance signifies the initiation and beginning of what usually becomes a robust pro-inflammatory signaling response highly detrimental to homeostatic brain cell structure and function. Such pathways are known to contribute to inflammatory neurodegeneration in central nervous system (CNS) cells of many types, leading to brain cell atrophy and progressive cell death [1–10]. Both mercury and aluminum have been shown to induce pro-inflammatory signaling towards the genetic apparatus of neurons at physiologically realistic ambient nanomolar (nM) levels [11–16]. It appears that of all of the biological targets in cells the genetic apparatus are particularly sensitive to both mercury and aluminum’s deleterious actions. For example, mercury interferes with, and mis-regulates, components of transcription such as adenosine triphosphate (ATP) generation, changes transcription rates and strongly interferes with DNA repair mechanisms [17–19]. Similarly aluminum (as sulfate) strongly alters the rates of transcription from neuron specific genes and much of this is accomplished: (i) in part through the up-regulation of the NF-kB (p50/p65) complex; (ii) by the direct up-regulation of deleterious pro-inflammatory genes; and (iii) by the up-regulation of microRNAs and subsequent down-regulation of brain essential and often neuronal-specific brain genes [15,16,19–23].

In these experiments we analyzed the effects of aluminum sulfate and mercury sulfate, either alone or together, on their ability to induce inflammatory signaling in human neuronal-glial (HNG) cells in primary cells, a neuronal-glial cell co-culture often used to analyze pathogenic disease mechanisms such as inflammatory neurodegeneration as a precursor to Alzheimer’s disease (AD) [14–16,24]. The evolution of the pro-inflammatory transcription factor NF-kB (p50/p65) complex was used as a neurobiological indicator of the onset of pathogenic inflammation. This is the first report on the neurotoxic effects of aluminum sulfate and/or mercury sulfate on the initiation of inflammatory neurodegeneration in human brain cells. The results show a potent up-regulation of inflammatory signaling by both mercury and aluminum and a significant synergism when they are both applied together. This work has both high neurotoxicological and environmental relevance since an up-regulation in inflammatory signaling is an important precursor event to many common, progressive and lethal human neurological diseases such as AD, and both aluminum and mercury represent two important types of neurotoxins enriched in our environment and the biosphere in which humans live.

## Materials and methods

### Human neuronal-glial (HNG) cells in primary co-culture

**Figure 1:**
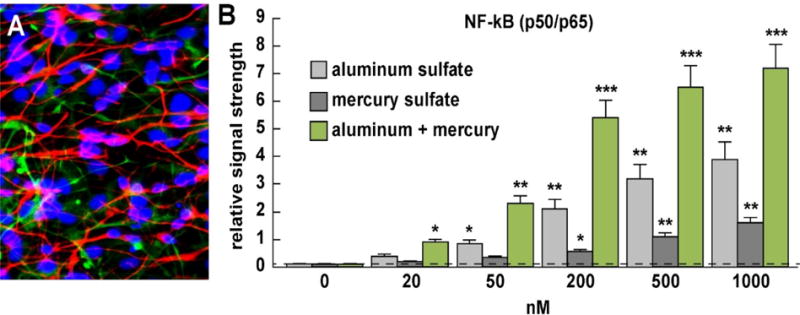
**(A)** primary human neuronal-glial (HNG) cells after ~2 weeks in primary co-culture; the cell density is approximately 75% neurons and 25% astroglia at ~70% confluency; human primary neuronal and glial “support” cell co-cultures are utilized because human neuronal cells do not culture well by themselves (28–32); HNG cells are triple stained; neuronal cells are stained with neuron-specific β-tubulin (red; λmax=690 nm), glial cells are stained with glial-specific glial fibrillary acidic protein (GFAP; green; λmax=525 nm), and nuclei are stained with DAPI/Hoechst 33258 stain (blue; λmax=470 nm); photo magnification ~30x; **(B)** ELISA results presented in bar graph format using an NF-κB (p65) transcription factor assay (Cat № 10007889 kit; Cayman Chemical, Ann Arbor MI USA; https://www.caymanchem.com/pdfs/10007889.pdf) to measure effects of incubation with 0, 20, 50, 200, 500 or 1000 nM ambient aluminum sulfate or mercury sulfate either alone or together; detection of NF-kB p65 is the equivalent of the NF-kB p50/p65 complex since the p65 subunit is always associated with the p50 subunit and is seldom found as a discrete single entity [1,2,59,60]; aluminum and mercury sulfate together exhibited synergistic induction of the pro-inflammatory transcription factor NF-kB (p65) under the conditions used; see text for further details); experiments were performed N=3 to 5 times per concentration analyzed; the background reading of 0.1 was established at ‘0 nM’ on the ‘x’ axis which was used for a comparison against all other levels; a dashed horizontal line at 0.1 has been added for ease of comparison; *p<0.05; **p<0.01, ***p<0.001, ANOVA)

HNG primary cells, cryopreserved at first passage were obtained from commercial sources and cultured according to the manufacturer’s instructions (Lonza PT-2599, Lonza Cell Systems, Allendale, NJ, USA or Cell Systems, ACBRI 376, Kirkland, WA, USA). HNG cells tested negative for HIV-1, HBV, HCV, mycoplasma, bacteria, yeast and fungi at source; HNG cells have been extensively used for studies on the effects of neurotoxins on brain gene expression; they exhibit particular neuronal and astroglial cell markers including glial fibrillary acidic protein (GFAP; glial-specific green stain; *λ*_max_=520 nm); neuron-specific β-tubulin III (βtubIII; red staining; *λ*_max_=690 nm); and a nuclear stain (DAPI; blue staining; *λ*_max_=470 nm) (Figure 1A). Briefly, HNG cells were maintained as free-floating aggregates (neurospheres) in 75 cm^2^ uncoated plastic flasks in neural progenitor maintenance media (NPMM; Lonza CC-3209) supplemented with human recombinant fibroblast growth factor (rhFGF), epidermal growth factor [rhEGF]), neural survival factor-1 [NSF-1] (Lonza CC-4242) and gentamicin/amphotericin-B (Lonza GA-1000). Differentiation was induced by plating these neurospheres onto 8-well glass chamber-slides pre-coated with poly-L-ornithine (an amino acid polymer used as substratum to improve HNG cell adhesion); cells were kept at 37°C in a humidified 5% CO_2_ atmosphere incubator at all times. The differentiation media (Lonza CC-4242) was free of growth factors but contained NSF and gentamicin/amphotericin-B, 25 ng/ml of brain-derived neurotrophic factor (BDNF) and 1% of fetal bovine serum (FBS). Upon deprivation of growth factors neurospheres began to attach to the well bottoms and next migrated out to form a co-culture of human neurons and glial cells (HNG). HNG cells were used 2 weeks after induction of differentiation; HNG cells initially contained about 5 × 10^5^ cells/ml volume and were cultured to about ~70% confluency in HNG cell medium as described in detail [14–16,24–32] (Figure 1A).

### Neurotoxic reagents – aluminum sulfate and mercury sulfate

#### Aluminum sulfate

Ultrapure aluminum sulfate [Al₂(SO₄)₃; aluminum sulfate anhydrous, 99.99%; CAS #: 10043-01-3; molecular weight 342.15 g/mol; solubility in water 364 g/l; total metal impurities: 0.01% max; commonly used as food additive and water purification agent; http://www.generalchemical.com/assets/pdf/Dry_Alum_Food_Grade_PDS.pdf; LD50 for aluminum sulfate in mice ~980 mg Al/kg) was purchased from Alfa Aesar – Thermo Fisher Scientific (Cat No. 44563-06) and solubilized according to the manufacturer’s instructions (https://www.alfa.com/en/catalog/044563/). From aluminum sulfate stock solutions 0, 20, 50, 200, 500 and 1000 nM solutions of NPMM containing aluminum sulfate were added to HNG cultures and incubated for 24 hours, at which time total HNG cellular nuclear proteins were analyzed for NF-κB (p65) abundance. Total nuclear proteins were extracted using a CellLytic™ NuCLEAR™ Extraction Kit (Product No. NXTRACT; https://www.sigmaaldrich.com/catalog/product/SIGMA/NXTRACT?lang=en&region=US) and analyzed as previously described [20,24].

#### Mercury sulfate

Mercury (II) sulfate (HgSO_4_; mercuric sulfate; CAS number 7783-35-9; molecular weight 296.65; p.a., ACS reagent, ≥99%) was purchased as Sigma-Aldrich Puriss (Cat. No 31013; Sigma-Aldrich; Millipore Sigma; St Louis MO, USA); mercury (II) sulfate is a highly neurotoxic odorless solid that forms white granules or crystalline powder is sparingly soluble in water (0.051 g/100 ml at 25℃); mercury (II) sulfate readily hydrolyzes in water, separating into the yellow mercuric subsulfate (Cat. No 31013; Sigma-Aldrich; Millipore Sigma;St Louis MO, USA). As for aluminum sulfate, from mercury (II) sulfate stock solutions 0, 20, 50, 200, 500 and 1000 nM solutions of NPMM containing mercury (II) sulfate were added to HNG cultures and incubated for 24 hours, at which time total HNG cell nuclear proteins were isolated using the NXTRACT methodology as above and analyzed for NF-κB (p65) abundance (https://www.sigmaaldrich.com/content/dam/sigma-aldrich/docs/Sigma/Bulletin/nxtractbul.pdf; https://www.epa.gov/sites/production/files/2016-09/documents/mercury-compounds.pdf).

### Assay for NF-kB (p50/p65) using ELISA

We used Cayman’s NF-κB (p65) transcription factor immunoabsorbant ELISA assay kit (Cat. No10007889; Cayman Chemical, Ann Arbor MI, USA), a non-radioactive, extremely sensitive method for detecting specific transcription factor DNA binding activity in nuclear extracts and whole cell lysates (detection limit 0.056 ng/mL). Note that p65 is seldom found as a single molecular entity in cells but is always complexed with NF-kB p50 forming the NF-kB (p50/p65) heterotypic dimer also known as RELA; virtually all of the NF-kB (p65) subunit is located in the NF-kB (p50/p65) complex [22–24,28]. In this assay a specific double stranded DNA (dsDNA) sequence containing the NF-kB (p50/p65) response element is immobilized and NF-kB (p50/p65) contained in a nuclear extract binds specifically to the NF-κB response element; subsequently NF-kB (p50/p65)) is detected by addition of specific primary antibody directed against NF-κB (p65) and a secondary antibody conjugated to HRP is added to provide a sensitive colorimetric readout at ~450 nm. This transcription factor assay detects human NF-κB (p65) and will not cross-react with any other NF-κB subunits; procedures have been previously described in detail according to the manufacturer’s instructions [23,24].

### Statistical analysis, integrated bioinformatics analysis and data interpretation

For NF-kB (p50/p65) abundance analysis all statistical procedures were analyzed using (*p*, analysis of variance (ANOVA) a two-way factorial analysis of variance using algorithms and/or procedures in the SAS language (Statistical Analysis Institute, Cary, NC, USA) and as previously described [24–34]. In the results *p*-values of less than 0.05 (ANOVA) were statistically significant. All NF-kB (p50/p65) abundance data were collected and analyzed using Excel 2016 (Office 365) algorithms (Microsoft Corporation, Redmond WA, USA); all figures were generated using Adobe Illustrator CC 2015 and Photoshop CC version14.0 (Adobe Corporation, San Jose, CA, USA).

## Results

Human neuronal-glial (HNG) cells in primary culture are shown in Figure 1A at about ~70% confluency and are comprised as approximately 75% neurons (red stained) cells and 25% astroglial (green stained) cells; pure human neuronal cells do not culture well by themselves and require the presence of astroglial cells for both nutritive and biophysical support [4,16,24,28,34]. Figure 1B shows the results in bar graph format of NF-kB (p65) evolution in response to treatment of 0, 20, 50, 200, 500 and 1000 nM aluminum sulfate and mercury sulfate incubated with HNG cells either alone or incubated together at the same concentration. Interestingly, we observed a classic dose-response profile for NF-kB (p65) evolution after 20, 50 and 200 nM aluminum and mercury sulfate were added to HNG cells; for example at 20 nM aluminum sulfate or mercury sulfate induced NF-kB (p65) about ~4-fold and ~2-fold above background; together ~20 nM aluminum and mercury sulfate induced NF-kB about ~9-fold above background. Similarly 50 nM aluminum sulfate or mercury sulfate induced NF-kB (p65) about ~8.5-fold and ~3.5-fold above background; together 50 nM aluminum and mercury sulfate induced NF-kB about 23-fold above background; and 200 nM aluminum sulfate or mercury sulfate induced NF-kB (p65) about ~21-fold and ~5.6-fold above background; together 200 nM aluminum and mercury sulfate induced NF-kB about 54-fold above background with very high significance (Figure 1B). Hence, inspection of these NF-kB (p65) induction events indicated (i) a significant increase of NF-kB (p65) induction after aluminum sulfate or mercury sulfate incubation; and (ii) a significant synergistic induction of NF-kB (p65) when both neurotoxic metals were added together. While 500 and 1000 nM ambient aluminum sulfate or mercury sulfate added, either alone or together continued to show these robust trends in NF-kB (p65) induction and a synergistic effect when both neurotoxic metals were added, the responses at these higher doses (500 and 1000 nM) were not in line with NF-kB (p65) induction at lower concentrations (20, 50 and 200 nM). This concentration effect of aluminum neurotoxicity has been previously reported where relatively more robust effects were actually observed at lower ambient concentrations than higher ones -and in the current experiments this may be due to the HNG plasma membrane barrier adaptation to aluminum exposure, the saturation of aluminum transport into cultured brain cells or other related off-target effects (see below) [22,35–38].

## Discussion

### Mercury exposure and daily intake

Naturally occurring inorganic mercury, with an average abundance of just 6.7×10^−6^ per cent of the earth’s crust is widely disseminated by natural processes such as volcanic activity and climatological effects, however the use of mercury in industrial processes has significantly increased in the last 70 years [39,40]. Mercury is currently used in the electrolytic production of chlorine and caustic soda, in electrical appliances (arc rectifiers, mercury batteries and lamps), in industrial and scientific switches, thermometers and barometers. Mercury is compounded for use as fungicides, antiseptics, preservatives, pharmaceuticals, electrodes, analytical reagents, dental amalgams, vaccine and venom preservatives as well as in a surprisingly large number of ethnic and folk remedies [41–43]. Overall, however, mercury’s general use has been decreasing because of environmental concerns and the realization of its potent neurotoxicity and genotoxicity. Mercury levels in air range from 2–10 ng/m^3^; the concentration of mercury in drinking-water is almost the same as in rain, with an average of about 25 ng/L; and the average daily intake of mercury from food is in the range 2–20 μg. Interestingly, only about 7–15% of ingested mercury in food is actually absorbed into the human body. The methylation of inorganic mercury under suboxic or anoxic conditions to yield methylmercury (MeHg) is an important toxifying process and occurs in in soils, sediments and in both fresh and sea waters via the actions of microbes, and especially methylating bacteria [40–44]. *Pseudomonas*, a genus of the Gram-negative *Gammaproteobacteria* belonging to the bacterial family *Pseudomonadaceae*, for example, resident in soils and aquatic organism microbiomes, can methylate mercury under aerobic conditions [43]. Methylmercury is far more toxic than inorganic mercury and environmental levels of methylmercury depend on the balance between bacterial methylation and demethylation. Certain species of marine animals such as the mackerel (*Scomber scombrus*), swordfish (*Xiphias gladius*), shark (*Carcharodon carcharias*) and tuna (*Thunnus alalunga*), and plants such as corn (*Zea mays)* and sugar cane (*Saccharum officinarum*) are natural mercury ‘biomagnifiers’; these organisms concentrate environmental mercury or methylmercury as a normal consequence of their growth [41–43].

In the current experiments the proportion of mercury in the form of MeHg was not determined, and its determination is both dynamic and variable and involves extremely toxic, hazardous and complex solution chemistries. All forms of ingested mercury, including MeHg, impairs cellular functions by altering the secondary, tertiary and quaternary structure of proteins through its high affinity and binding with sulfhydryl and selenohydryl groups such as those found in the sulfur-containing amino acids cysteine and methionine [40–42]. While mercury can potentially impair the function of any tissue, organ, cell, subcellular or nuclear structure, a major target of mercury is the central nervous system (CNS) and the genetic material located therein [39–44]. Mercuric salts at nM concentrations have been shown to induce cellular cytotoxicity, neurotoxicity, genotoxicity and ROS generation while increasing secretion of neurotoxic amyloid-beta peptides in cultured brain cells, thereby contributing to the pathophysiological mechanisms which are characteristic of the AD process [43,44]. Mercury additionally targets the peripheral nervous system (PNS), the immune and inflammatory system, and endocrine, kidney, muscle and skin functions that include a mercury-mediated contact dermatitis [39,41–43].

### Aluminum exposure and intake

Aluminum, the most abundant metallic element in the environment and constituting about 8% (w/v) of the Earth’s crust occurs naturally as hydroxides, oxides and silicates, and combines with other elements, such as sodium and fluoride, and as complexes with organic matter. Aluminum sulfate [Al_2_(SO_4_)_3_] is a common additive to drinking water worldwide as a ‘clarifying agent’. Aluminum enters the earth’s atmosphere as a major constituent of atmospheric particulates originating from agricultural activities, coal combustion, natural soil erosion, surface mining or volcanic eruptions. Concentrations of atmospheric aluminum ‘dust’ show widespread temporal and spatial variations and airborne aluminum range from 0.0005 μg/m^3^ over Antarctica to more than 1 μg/m^3^ in industrialized areas [45–48]. Natural aluminum concentrations in waters vary significantly depending on various physicochemical, mineralogical and climatological factors. For example, dissolved aluminum in water with near-neutral pH values typically range from 0.001 to 0.05 mg/L however these levels can rise to 0.5–1 mg/L in more acidified waters or in water enriched in organic matter; in the extreme acidity of waters affected by acid mine drainage, dissolved aluminum concentrations of up to 90 mg/L have been reported [45–48].

Aluminum is naturally present in foods, from the use of aluminum-containing food additives or from aluminum cookware, utensils and wrappings; foods naturally high in aluminum include potatoes, spinach, tea, processed dairy products such as cheese, flour and infant formula. The average human intake of aluminum in the USA is about 7.1–8.2 mg/day; these represent the major route of aluminum exposure for the general population, excluding persons who regularly ingest aluminum-containing antacids and buffered analgesics, for whom intakes may be as high as 5 gm/day [49–51]. In biological systems including transgenic animal models for AD (TgAD) aluminum is an extremely potent inducer of both reactive oxygen species (ROS) and the pro-inflammatory transcription factor NF-kB p50/p65 complex [14–16,22].

### Aluminum and mercury exposure together

Very little research exists on the combined exposure of aluminum and mercury together; intake of water from highly acidified lakes (increased aluminum exposure) in areas of high industrial activities such as battery production, mining, smelting, pulp-and-paper production and the mordanting of textiles (increased mercury exposure) may provide a ‘*perfect storm’* for combined aluminum+mercury intake and the synergistic pro-inflammatory and neurotoxicological effects of simultaneous exposure to both of these noxious metals [35,36,51–56]. Certain medicinal compounds including some vaccines, skin test antigens, anti-venoms (antivenins), ophthalmic and nasal products and tattoo inks contain the organomercury compound Thimerasol (Merthiolate; Eli Lily) as a well-established antiseptic and antifungal preserving agent; for example, vaccines administered with aluminum hydroxide adjuvants might also expose individuals to both aluminum- and mercury-based neurotoxins simultaneously [21,52–57]. Interestingly, aluminum can form an amalgam in solution with mercury for use as a chemical reagent to reduce compounds, such as the reduction of imines to amines, or as an experimental component of dental amalgam [57,58].

### NF-kB and inflammatory signaling

The NF-kB family of transcription factors is comprised of several structurally related, pleiotrophic homo- or heterodimeric DNA-binding complexes including NF-kB (p50/p65); the heterodimeric NF-kB p50-p65 complex appears to be most abundant in the human CNS [59–61]. NF-kB family members are responsible for up-regulating dozens of genes including those expressing the pro-inflammatory cytokines, chemokines, immuno-receptors, cell adhesion molecules and microRNAs [1–6,59–62]. Because of this NF-kB signaling is often a central mediator of the immune and inflammatory response; its activation represents the common endpoint of a series of signal transduction events initiated by a vast array of stimuli related to many biological processes such as apoptosis, cell growth, differentiation, innate-immunity, inflammation, tumor cell growth and the presence of ROS generated by neurotoxic metals in the environment [59,60]. NF-kB (p50/p65) dimeric complexes are typically held in the cytoplasm in an inactive state complexed with an NF-kB inhibitor (I-kappa-B; IkB) protein; IkB is next typically phosphorylated by IkB kinases (IKKs) in response to different activators such as ROS from different sources and is subsequently degraded, thus liberating an active NF-kB (p50/p65) complex. These next translocate to the nucleoplasm where they recognize and bind to NF-kB features of DNA, typically in the upstream promoter DNA and activate large numbers of NF-kB-sensitive genes [1–6,59–62]. The 551-amino acid NF-kB p65 subunit, also known as RELA or NFKB3, is encoded from a 1473 nucleotide gene located on human chr 1q13 and contains a N-terminal REL-homology domain (RHD) and a C-terminal transactivation domain (TAD); the RHD is involved in the actual DNA binding, dimerization and NF-κB/REL inhibitor interaction [1,2,59,60]. NF-kB p65 (RELA) is expressed alongside NF-kB p50 in various cell types, including epithelial, endothelial and neuronal tissues. Interestingly, as our current results indicate, the NF-kB (p65) complex is significantly up-regulated by both aluminum sulfate and mercury sulfate, and synergistically by aluminum sulfate and mercury sulfate together, and a ROS-type activation mechanism is suspected [14–16] (Figure 1B).

### Aluminum and mercury dietary exposure: potential impact on human disease

A number of recent studies underscore the impact of aluminum and mercury in our everyday diet and their potential contribution to neurotoxicity and human disease. Aluminum mobilization into our environment and into our diet from multiple sources and via multiple uptake mechanisms has been extensively reviewed [14–17,21,22,46,48,50–56,63]. Dietary composition and complexity are important for neurotoxic metal intake, for example total blood mercury levels may be influenced by the dietary intake of highly processed foods, and lower inorganic mercury levels in the blood are associated with lower fasting glucose levels [64]. Water soluble forms of mercury have been shown to appear in the blood and urine of children exposed to mercury-contaminated waters via both cooking and drinking [65]. Higher blood lead and mercury levels have been correlated with the severity of social and cognitive impairment in autistic children [66]. Gut microbiota abundance, speciation and complexity, in part based on dietary intake and dietary factors such as fructose and fiber intake, has considerable potential to modulate the metabolism of methylmercury [67–69]. Interestingly, female C57BL/6J mice fed Western-style diets supplemented with high fructose exhibited altered gastrointestinal (GI) tract permeability and GI barrier dysfunction that was correlated with an increased systemic exposure to neurotoxic metals [70]. It is clear that there are a multitude of biochemical and physiological pathways and mechanisms that modulate neurotoxic metal uptake from the environment via dietary factors, and these are capable of exacerbating both neurotoxicity and genotoxicity, and the initiation and development of human disease.

## Conclusions

A growing body of investigative evidence continues to implicate environmental and dietary aluminum and mercury uptake to many aspects of human physiological and in particular, neurobiological dysfunction. While the current research work describes relatively simple and straight-forward *in vitro* experiments, they are certainly informative as to the role of aluminum and/or mercury (as sulfates) either alone or together in human brain cells. Primary HNG cells are rather challenging and time-consuming to culture, however, they are exactly the same brain cell types targeted by the inflammatory neurodegeneration that characterizes age-related and lethal neurological disorders such as AD. Both aluminum and mercury sulfates, as do most other aluminum and mercury compounds, have especially complex aqueous chemistries and their abundance, complexity and speciation depend on their metal ion concentration, aqueous pH, solution temperature, the presence of other biological ligands, host genetics and epigenetics, and other highly interactive chemical, biophysical, biological and environmental factors.

In summary, the experimental work in this paper provides 3 new observations: (i) that nanomolar quantities of either aluminum sulfate [Al₂(SO₄)₃] or mercury (II) sulfate (HgSO_4_; mercuric sulfate) significantly induce the pro-inflammatory NF-kB (p65) activator complex in HNG cells in primary culture; (ii) that aluminum sulfate and mercury sulfate when added together exhibit a remarkable and significant synergism in their activation of the NF-kB (p65) complex to manifold than that which each metal sulfate could induce by itself; and (iii) that neurotoxic metal sulfates obtainable via our environment or diet are particularly potent in inducing the pro-inflammatory NF-kB (p65) activator complex. This inducible precursor to pathogenic gene expression programs is known to drive inflammatory neurodegeneration and progressive age-related functional decline, thus upsetting the normal, homeostatic operation of the human CNS.

## References

[1] Gilmore TD (1999). The Rel/NF-kappaB signal transduction pathway: introduction. Oncogene.

[2] Pahl HL (1999). Activators and target genes of Rel/NF-kappaB transcription factors. Oncogene.

[3] Gilroy DW, Lawrence T, Perretti M, Rossi AG (2004). Inflammatory resolution: new opportunities for drug discovery. Nat Rev Drug Discov.

[4] Lukiw WJ (2012). NF-кB-regulated micro RNAs (miRNAs) in primary human brain cells. Exp Neurol.

[5] Lukiw WJ (2012). NF-κB-regulated, proinflammatory miRNAs in Alzheimer’s disease. Alzheimers Res Ther.

[6] Franceschi C, Ottaviani E (1997). Stress, inflammation and natural immunity in the aging process: a new theory. Aging (Milano).

[7] Du X, Wang X, Geng M (2018). Alzheimer’s disease hypothesis and related therapies. Transl Neurodegener.

[8] Stephenson J, Nutma E, van der Valk P, Amor S (2018). Inflammation in CNS Neurodegenerative Diseases. Immunology.

[9] Kempuraj D, Thangavel R, Selvakumar GP, Zaheer S, Ahmed ME (2017). Brain and peripheral atypical inflammatory mediators potentiate neuroinflammation and neurodegeneration. Front Cell Neurosci.

[10] Franceschi C, Bonafè M, Valensin S, Olivieri F, De Luca M (2000). Inflammaging. An evolutionary perspective on immunosenescence. Ann N Y Acad Sci.

[11] Lu X, Xiang Y, Yang G, Zhang L, Wang H (2017). Transcriptomic characterization of zebrafish larvae in response to mercury exposure. Comp Biochem Physiol C Toxicol Pharmacol.

[12] Monroe RK, Halvorsen SW (2006). Mercury abolishes neurotrophic factor-stimulated Jak-STAT signaling in nerve cells by oxidative stress. Toxicol Sci.

[13] Habibi L, Shokrgozar MA, Tabrizi M, Modarressi MH, Akrami SM (2013). Mercury specifically induces LINE-1 activity in a human neuroblastoma cell line. Mutat Res Genet Toxicol Environ Mutagen.

[14] Pogue AI, Jones BM, Bhattacharjee S, Percy ME, Zhao Y (2012). Metal-sulfate induced generation of ROS in human brain cells: detection using an isomeric mixture of 5- and 6-carboxy-2′,7′-dichlorofluorescein diacetate (carboxy-DCFDA) as a cell permeant tracer. Int J Mol Sci.

[15] Lukiw WJ, Bhattacharjee S, Zhao Y, Pogue AI, Percy ME (2012). Generation of reactive oxygen species (ROS) and pro-Inflammatory signaling in human brain cells in primary culture. J Alzheimers Dis Parkinsonism.

[16] Lukiw WJ, Pogue AI (2007). Induction of specific micro RNA (miRNA) species by ROS-generating metal sulfates in primary human brain cells. J Inorg Biochem.

[17] Pogue AI, Jaber V, Zhao Y, Lukiw WJ (2017). Systemic inflammation in C57BL/6J mice receiving dietary aluminum sulfate; up-regulation of the pro-inflammatory cytokines IL-6 and TNFα, C-reactive protein (CRP) and miRNA-146a in blood serum. J Alzheimers Dis Parkinsonism.

[18] Dixit V, Bini E, Drozda M, Blum P (2004). Mercury inactivates transcription and the generalized transcription factor TFB in the archaeon *Sulfolobus solfataricus*. Antimicrob Agents Chemother.

[19] Gadhia SR, Calabro AR, Barile FA (2012). Trace metals alter DNA repair and histone modification pathways concurrently in mouse embryonic stem cells. Toxicol Lett.

[20] Lukiw WJ, LeBlanc HJ, Carver LA, McLachlan DR, Bazan NG (1998). Run-on gene transcription in human neocortical nuclei. Inhibition by nanomolar aluminum and implications for neurodegenerative disease. J Mol Neurosci.

[21] Schofield K (2017). The metal neurotoxins: An important role in current human neural epidemics?. Int J Environ Res Public Health.

[22] Pogue AI, Lukiw WJ (2016). Aluminum, the genetic apparatus of the human CNS and Alzheimer’s disease (AD). Morphologie.

[23] Cayman Chemicals https://www.caymanchem.com/pdfs/10007889.pdf.

[24] Cui JG, Li YY, Zhao Y, Bhattacharjee S, Lukiw WJ (2010). Differential regulation of interleukin-1 receptor-associated kinase-1 (IRAK-1) and IRAK-2 by microRNA-146a and NF-κB in stressed human astroglial cells and in Alzheimer disease. J Biol Chem.

[25] Bhattacharjee S, Lukiw WJ (2013). Alzheimer’s disease and the microbiome. Front Cell Neurosci.

[26] Lukiw WJ (2016a). Bacteroides fragilis lipopolysaccharide and inflammatory signaling in Alzheimer’s disease. Front Microbiol.

[27] Lukiw WJ (2016b). The microbiome, microbial-generated proinflammatory neurotoxins, and Alzheimer’s disease. J Sport Health Sci.

[28] Zhao Y, Bhattacharjee S, Jones BM, Hill J, Dua P (2014). Regulation of neurotropic signaling by the inducible, NF-kB-sensitive miRNA-125b in Alzheimer’s disease (AD) and in primary human neuronal-glial (HNG) cells. Mol Neurobiol.

[29] Zhao Y, Cong L, Jaber V, Lukiw WJ (2017a). Microbiome-derived lipopolysaccharide enriched in the perinuclear region of Alzheimer’s disease brain. Front Immunol.

[30] Zhao Y, Jaber V, Lukiw WJ (2017b). Secretory products of the human GI tract and their potential impact on Alzheimer’s disease (AD): detection of lipopolysaccharide (LPS) in AD Hippocampus. Front Cell Infect Microbiol.

[31] Zhao Y, Calon F, Julien C, Winkler JW, Petasis NA, Lukiw WJ (2011). Docosahexaenoic acid-derived neuroprotectin D1 induces neuronal survival via secretase- and PPARγ-mediated mechanisms in Alzheimer’s disease models. PLoS One.

[32] Clement C, Hill JM, Dua P, Culicchia F, Lukiw WJ (2016). Analysis of RNA from Alzheimer’s disease post-mortem brain tissues. Mol Neurobiol.

[33] Dendooven T, Luisi BF (2017). RNA search engines empower the bacterial intranet. Biochem Soc Trans.

[34] Zhao Y, Lukiw WJ (2018). Microbiome-mediated up-regulation of miRNA-146a in sporadic Alzheimer’s disease (AD). Front Neurol.

[35] Drobyshev EJ, Solovyev ND, Gorokhovskiy BM, Kashuro VA (2018). Accumulation patterns of sub-chronic aluminum toxicity model after gastrointestinal administration in rats. Biol Trace Elem Res.

[36] Li Y, Jiao Q, Xu H, Du X, Shi L (2017). Biometal dyshomeostasis and toxic metal accumulations in the development of Alzheimer’s disease. Front Mol Neurosci.

[37] Crépeaux G, Eidi H, David MO, Baba-Amer Y, Tzavara E (2017). Non-linear dose-response of aluminium hydroxide adjuvant particles: Selective low dose neurotoxicity. Toxicology.

[38] Yokel RA, Unrine JM (2017). Aluminum and phthalates in calcium gluconate: contribution from glass and plastic packaging. J Pediatr Gastroenterol Nutr.

[39] World Health Organization(WHO) report – Policy paper 2005.

[40] Barbalace KL (2017). EnvironmentalChemistry.com; Periodic Table of the Elements – mercury.

[41] Livestrong.com (2018). Side effects of mercury in the diet.

[42] Bernhoft RA (2012). Mercury toxicity and treatment: a review of the literature. J Environ Public Health.

[43] Liem-Nguyen V (2016). Determination of mercury chemical speciation in the presence of low molecular mass thiols and its importance for mercury methylation. Doctoral thesis, Umeå.

[44] Olivieri G, Brack C, Müller-Spahn F, Stähelin HB, Herrmann M (2000). Mercury induces cell cytotoxicity and oxidative stress and increases beta-amyloid secretion and tau phosphorylation in SHSY5Y neuroblastoma cells. J Neurochem.

[45] Barbalace KL (2017). EnvironmentalChemistry.com; Periodic Table of the Elements – aluminum.

[46] Lenntech (2018). Aluminum and water: reaction mechanisms, environmental impact and health effects.

[47] Waters AS, Webster-Brown JG (2013). Assessing aluminum toxicity in streams affected by acid mine drainage. Water Sci Technol.

[48] World Health Organization (WHO) report–Policy paper 2005.

[49] Willhite CC, Karyakina NA, Yokel RA, Yenugadhati N, Wisniewski TM (2014). Systematic review of potential health risks posed by pharmaceutical, occupational and consumer exposures to metallic and nanoscale aluminum, aluminum oxides, aluminum hydroxide and its soluble salts. Crit Rev Toxicol.

[50] Klotz K, Weistenhöfer W, Neff F, Hartwig A, van Thriel C (2017). The health effects of aluminum exposure. Dtsch Arztebl Int.

[51] Bondy SC (2016). Low levels of aluminum can lead to behavioral and morphological changes associated with Alzheimer’s disease and age-related neurodegeneration. Neurotoxicology.

[52] Frisbie SH, Mitchell EJ, Sarkar B (2015). Urgent need to reevaluate the latest World Health Organization guidelines for toxic inorganic substances in drinking water. Environ Health.

[53] Margaret E, Sears SJ (2012). Genius environmental determinants of chronic disease and medical approaches: recognition, avoidance, supportive therapy, and detoxification. J Environ Public Health.

[54] von Stackelberg K, Guzy E, Chu T, Henn BC (2015). Exposure to mixtures of metals and neurodevelopmental outcomes: a review risk anal. Risk Anal.

[55] Carpenter DO, Arcaro K, Spink DC (2002). Understanding the human health effects of chemical mixtures. Environ Health Perspect.

[56] Flora SJ (2009). Structural, chemical and biological aspects of antioxidants for strategies against metal and metalloid exposure. Oxid Med Cell Longev.

[57] Briody RG, Cuevas EA (1971). Alloys based on mercury; aluminum amalgam.

[58] Thomas GP (2013). Amalgam-chemical composition, mechanical properties, and common applications.

[59] Lukiw WJ, Bazan NG (1998). Strong nuclear factor-kB-DNA binding parallels cyclooxygenase-2 gene transcription in aging and in sporadic Alzheimer’s disease superior temporal lobe neocortex. J Neurosci Res.

[60] Freitas RHCN, Fraga CAM (2018). NF-κB-IKKβ pathway as a target for drug development: realities, challenges and perspectives. Curr Drug Targets.

[61] Kruck TPA, Percy ME, Lukiw WJ (2008). Metal sulfate-mediated induction of pathogenic genes and repression by phenyl butyl nitrone and Feralex-G. Neuroreport.

[62] Pires BRB, Silva RCMC, Ferreira GM, Abdelhay E (2018). NF-kB: Two sides of the same coin. Genes (Basel).

[63] Pogue AI, Lukiw WJ (2014). The mobilization of aluminum into the biosphere. Front Neurol.

[64] Dufault R, Berg Z, Crider R, Schnoll R, Wetsit L (2015). Blood inorganic mercury is directly associated with glucose levels in the human population and may be linked to processed food intake. Integr Mol Med.

[65] Arcega-Cabrera F, Fargher LF, Oceguera-Vargas I, Noreña-Barroso E, Yánez-Estrada L (2017). Water consumption as source of arsenic, chromium, and mercury in children living in rural Yucatan, Mexico: blood and urine levels. Bull Environ Contam Toxicol.

[66] Alabdali A, Al-Ayadhi L, El-Ansary A (2014). A key role for an impaired detoxification mechanism in the etiology and severity of autism spectrum disorders. Behav Brain Funct.

[67] Rothenberg SE, Keiser S, Ajami NJ, Wong MC, Jonathan Gesell J (2016). The role of gut microbiota in fetal methylmercury exposure: insights from a pilot study. Toxicol Lett.

[68] Jang C, Hui S, Lu W, Cowan AJ, Morscher RJ (2018). The small intestine converts dietary fructose into glucose and organic acids. Cell Metab.

[69] Lukiw WJ (2016). The microbiome, microbial-generated pro-inflammatory neurotoxins, and Alzheimer’s disease. J Sport Health Sci.

[70] Volynets V, Louis S, Pretz D, Lang L, Ostaff MJ (2016). Intestinal barrier function and the gut microbiome are differentially affected in mice fed a Western-style diet or drinking water supplemented with fructose. J Nutr.

